# Assessing the Ability of Generative Adversarial Networks to Learn Canonical Medical Image Statistics

**DOI:** 10.1109/TMI.2023.3241454

**Published:** 2023-06-01

**Authors:** Varun A. Kelkar, Dimitrios S. Gotsis, Frank J. Brooks, Prabhat KC, Kyle J. Myers, Rongping Zeng, Mark A. Anastasio

**Affiliations:** Department of Electrical and Computer Engineering, University of Illinois at Urbana–Champaign, Urbana, IL 61801 USA; Department of Electrical and Computer Engineering, University of Illinois at Urbana–Champaign, Urbana, IL 61801 USA; Department of Bioengineering, University of Illinois at Urbana–Champaign, Urbana, IL 61801 USA; Center for Devices and Radiological Health, Food and Drug Administration, Silver Spring, MD 20993 USA; Center for Devices and Radiological Health, Food and Drug Administration, Silver Spring, MD 20993 USA; Center for Devices and Radiological Health, Food and Drug Administration, Silver Spring, MD 20993 USA; Department of Bioengineering, University of Illinois at Urbana–Champaign, Urbana, IL 61801 USA

**Keywords:** Generative models, generative adversarial networks, stochastic image models, objective image quality assessment

## Abstract

In recent years, generative adversarial networks (GANs) have gained tremendous popularity for potential applications in medical imaging, such as medical image synthesis, restoration, reconstruction, translation, as well as objective image quality assessment. Despite the impressive progress in generating high-resolution, perceptually realistic images, it is not clear if modern GANs reliably learn the statistics that are meaningful to a downstream medical imaging application. In this work, the ability of a state-of-the-art GAN to learn the statistics of canonical stochastic image models (SIMs) that are relevant to objective assessment of image quality is investigated. It is shown that although the employed GAN successfully learned several basic first- and second-order statistics of the specific medical SIMs under consideration and generated images with high perceptual quality, it failed to correctly learn several per-image statistics pertinent to the these SIMs, high-lighting the urgent need to assess medical image GANs in terms of objective measures of image quality.

## I. INTRODUCTION

When developing improved medical imaging technologies it is important to objectively evaluate them with consideration of relevant clinical tasks [1], [2], [3], [4]. However, clinical trials of emerging imaging technologies are often impractical or infeasible [5], [6]. Hence, computer simulation studies [7] have been proposed as an alternative. In order to refine and assess any medical imaging technology via computer simulation, the nature and variability of the objects to-be-imaged must be accurately characterized. To this end, a variety of stochastic object models (SOMs) have been developed [5], [7] that enable simulation of random, and sufficiently realistic, digital objects that can be virtually imaged.

A generative model is a statistical model of an unknown data distribution that enables sampling from the data distribution via a learned representation. The model is trained directly on a large sample drawn from the data distribution [8]. Modern generative models typically learn a neural network-based mapping from a tractable distribution (for eg. a multivariate, independent, and identically distributed (i.i.d.) Gaussian distribution) to the intractable, high-dimensional object distribution of interest. This enables sampling from the unknown distribution, and provides the ability to perform inference. Deep generative models, such as generative adversarial networks (GANs), are being actively investigated for a variety of medical imaging applications that include image restoration [9], [10], image reconstruction [11], [12], [13], [14], image analysis [15], [16], image-to-image translation [17], data sharing [18] and objective image quality assessment [19].

Modern generative models, such as the StyleGAN and its successors [20], [21], [22], have yielded tremendous improvements in terms of the stability, controllability, diversity, and visual quality of generated images. However, state-of-the-art GANs trained on medical image datasets have been shown to produce images that look realistic, but nevertheless contain potentially impactful errors [18], [23], [24]. Therefore, in order for GANs to be safely used in medical imaging applications, they must be objectively evaluated [25].

Despite tremendous improvements in the quality of the images generated by a GAN, the question of whether or not a GAN correctly approximates the statistical features important to a medical imaging application remains largely unanswered. Mathematical summaries, such as the Wasserstein metric [26] and negative log-likelihood [27] are correlated with the fidelity of the trained GAN. However, a favorable value achieved by these measures does not guarantee that the GAN is useful for a particular medical imaging application. Perceptual measures such as the Frechet Inception distance (FID) have been widely employed but are agnostic to the downstream task a medical image GAN may be used for [28]. Furthermore, the above mentioned measures are ensemble measures. It has been shown that individual samples drawn from the GAN may contain impactful errors despite giving satisfactory ensemble measures [29]. Lastly, medical image distributions typically consist of multiple classes or modes. It has been shown that GANs may produce critical errors while producing images from a mode that is rarely seen during training [18].

The objectives of this study are to (1) assess the ability of a state-of-the-art GAN to learn canonical statistics of several stochastic image models (SIMs) that are relevant to medical imaging applications, and (2) to study how task-agnostic measures such as FID score compare with clinically meaningful measures identified for the canonical SIM under consideration. Three canonical SIMs were identified for use in this study: the modified clustered lumpy background model [30], the B-mode ultrasound speckle model [31] and the stylized two-dimensional (2D) VICTRE (S2V) model [7]. A state-of-the-art GAN architecture, namely Style-GAN2, was trained on images generated from these SIMs. Statistical quantities that are meaningful and relevant to the above SIMs were identified and computed from both the original images produced by use of the SIM as well as the images generated by the GAN. Summary measures computed from these statistical quantities were compared against the FID for the purpose of assessing the fidelity of the trained GAN. This work is an extension of a preliminary study conducted using an angiographic SIM [32].

## II. BACKGROUND

### A. Generative Adversarial Networks (GANs)

Generative adversarial networks (GANs) are a popular class of implicit generative models that are designed to sample from a data distribution. This is accomplished by learning to map a sample $z\in R^{k}$ from a lower dimensional, tractable data distribution $p_{z}$, such as the i.i.d. standard normal distribution, to a sample $f\in R^{n}$ from the high dimensional data distribution $p_{f}$. In GANs, two networks, namely a *generator network* $G\;:R^{k}\to R^{n}$ with parameters $\Theta_{G}$ and a *discriminator network* $D\;:R^{n}\to R$ with parameters $\Theta_{D}$ are jointly trained by approximately solving the following min-max optimization problem:

(1) $$\underset{\Theta_{G}}{min}\;\underset{\Theta_{D}}{max}\;E_{f∼p_{f}}\left[\ell \left(D_{\Theta_{D}}(f)\right)\right]+E_{z∼p_{z}}\left[\ell \left(1-D_{\Theta_{D}}\left(G_{\Theta_{G}}(z)\right)\right)\right],$$

where ℓ(⋅) is a utility function used to define the objective; for instance, a popular choice being ℓ(x)=log(x) [33]. The promise of a generative model such as a GAN comes from the fact that once trained, samples from the otherwise inaccessible high dimensional distribution $p_{f}$ can be obtained by sampling low dimensional vectors z, known as latent vectors, from $p_{z}$ and computing G(z). Thus, the GAN provides a tractable representation of $p_{f}$ that may find use in downstream applications in imaging science, such as image reconstruction [11], [14] and image quality assessment [19].

### B. Advanced GAN Training Strategies

Under prescribed theoretical conditions, minimizing the GAN training loss described in Eq. (1) is equivalent to minimizing the empirical Jensen-Shannon (JS) divergence between the true and the estimated probability distribution functions (PDFs) of the data [33]. However, in practice, GAN training is known to be unstable [34], [35] and several strategies have been proposed to improve stability. For example, the use of different learning rates and update frequencies for the generator and discriminator weights helps avoid the vanishing gradients problem for the generator and premature overfitting of the discriminator [33], [36]. Novel loss functions, such as in so-called Wasserstein GANs [26], also help in improving the training stability. Karras et al. [37] proposed a strategy for scaling GANs by use of *progressive training*. Here, both the generator and discriminator are trained on lower resolution images and are progressively grown to enable training on higher and higher resolution images. StyleGAN and its successor, StyleGAN2, introduce blocks of transformed latent vectors as inputs to different layers of a synthesis network at different resolutions. This enables control of features at different scales [20], [21]. Such improvements to the GAN architecture and training have cumulatively led to state-of-the-art performance in terms of diversity, controllability and realism of images generated. However, advancements in GAN technologies have have not be motivated by the need learn task-pertinent statistics associated with medical imaging applications.

### C. Evaluation of Generative Adversarial Networks

Modern GANs, such as the StyleGAN2 [21] have shown impressive performance in terms of the perceptual quality of the generated images, invertibility, and meaningful control over image semantics. However, evaluating the quality of the distribution learned by a generative model is an open problem [38]. Some measures directly estimate analytical quantities and distance metrics related to the image probability density function (PDF), such as the negative log-likelihood [27] or the Wasserstein metric [26]. Other measures such as the perceptual path-length [20] analyze the nature of the manifold learned by the GAN. Motivated by subjective perceptual assessment by humans [39], perceptual evaluation measures such as the Inception score (IS) and more commonly, the Fréchet Inception distance (FID) score are currently popular [28], [39]. In order to compute these scores, image features are first extracted using a pre-trained Inception network [40] and distance metrics on the extracted features are computed. The FID score has shown excellent agreement with subjective visual assessments by humans [28]. However, it is agnostic to the downstream task a medical image GAN may be used for. Additionally, it is an ensemble statistic, and hence could be blind to specific errors in high-order statistics of individual images [29].

The studies described below seek to assess the ability of medical image GANs to reproduce image statistics that are known to be useful for medical assessments of the simulated medical images produced by the SIM (henceforth referred to as “statistics pertinent to the SIM”). This work also studies how well traditional measures such as the FID correlate with these pertinent statistics. For the purpose of these assessments, the data distributions used to train the GAN needs to be carefully chosen as follows. First, clinically relevant SIMs that prescribe a mathematical procedure for generating images need to be identified. This allows for direct control over image properties of interest. For these SIMs, germane statistical quantities need to be identified. These tasks are described next.

### D. Canonical Stochastic Image Models

Stochastic models of simulated medical images have been developed in order to approximately capture the variability in medical image distributions [1], [5], [41]. Such stochastic image models (SIMs) have been established by developing a mathematical procedure for generating images that possess certain prescribed statistical properties. Examples of such SIMs include the lumpy background model [41], the clustered lumpy background (CLB) model [42], B-mode ultrasound speckle model [31], among others. Once a SIM is established, it can be used to model image statistics in virtual imaging trials [7].

In this work, SIMs corresponding to a modified clustered lumpy background model [30], the B-mode ultrasound speckle model [31] and a stylized 2D VICTRE breast phantom model were employed [7]. These were chosen because they have been employed previously for simulating realistic canonical medical images with different statistical properties [5], [7], [30]. Additionally, ample literature exists that describes the statistics relevant to diagnostic tasks for the image types associated with these SIMs [43], [44], [45]. Differently from the use of real medical images, simulated images from these SIMs provide the ability to examine the behavior of the GAN in a controlled setting in which there are no uncharacterized sources of variablity in the image data. Some salient details regarding the SIMs to be employed are provided next.

#### 1) Modified Clustered Lumpy Background (CLB) Model:

The CLB model was developed by Bochud et al. [42] for generating random backgrounds that resemble the image textures seen in mammography. In 2008, Castella, et al. proposed variations to the original CLB model so that the images from the model better resemble realistic mammographic textures as judged by human experts [30]. In addition to introducing oriented structures and long-range correlations, the authors proposed to adjust the parameters of the CLB model in order to improve the realism of the images generated. This was done by computing 17 different texture features on both the real mammographic regions of interest (ROIs) as well as images generated from the CLB model. These were used to formulate a loss function that was minimized by tuning the parameters of the CLB model.

#### 2) B-Mode Ultrasound Speckle (USS):

B-mode ultrasound speckle (USS) can be viewed as a random phasor sum of complex signals [31]. The received complex signal E is a radio frequency voltage output from an ultrasound transducer. It can be modeled as the sum of N complex signals with phases statistically independent uniformly distributed on [0,2π] [31]. The quantity N is the number of scatterers per resolution cell or equivalently the scatterers per number density (SND) times the resolution cell size. The resolution cell size is defined as the axial resolution (AR) times the lateral resolution (LR), given in [46], where the parameters are the frequency of the carrier $f_{c}$, the wave velocity v, the ratio between the focal distance and the length of the aperture (called the f - number) and the number of cycles within the full width half maximum in the spatial direction (FWHM) $N_{c}$. The USS SIM is modeled using the method proposed in [31] where the standard deviations of the 2-D Gaussian PSF are determined by the AR and LR.

If N is large, the resulting USS follows Gaussian statistics and is called fully developed speckle. In this case, the envelope |E| follows a Rayleigh distribution and thus the intensity $I=|E{|}^{2}$ follows an exponential distribution. If N is small then the resulting USS is called non-Gaussian speckle and its statistical properties are determined by N [31].

#### 3) The Stylized 2D VICTRE (S2V) Breast Phantom Model:

The US Food and Drug Administration’s (FDA) Virtual Imaging Clinical Trials for Regulatory Evaluation (VICTRE) initiative has produced a set of software tools for simulating random anthropomorphic phantoms of the human female breast [7]. These numerical breast phantoms (NBPs) are three dimensional (3D) voxelized maps. Each voxel in the NBPs is labeled by one of the following 10 tissues: fat, glandular tissue, skin, artery, vein, muscle, ligament, nipple and terminal duct lobular unit. Controlling the patient-specific input parameters such as breast type, size, shape, granularity and density, and setting the random seed number enables the generation of large ensembles of stochastic NBPs with realistic variation in breast anatomy, shape and fat-to-glandular tissue ratio. The VICTRE model is thus a general stochastic object model (SOM) that can be specialized to different imaging modalities by assigning the appropriate physical coefficients. In particular, by assigning X-ray linear attenuation coefficients to the various tissues in the NBPs and extracting 2D slices from the 3D phantom, a SIM can be obtained. The VICTRE software creates NBPs that correspond to four breast types identified by the American College of Radiology’s (ACR) Breast Imaging Reporting and Data System (BI-RADS) [47] and are distinguished by the amounts of fat and glandular tissue.

## III. NUMERICAL STUDIES

### A. SIM Training Data and GAN Training

#### 1) The CLB Model:

The following four parameter configurations of the modified CLB model were used in this study – (1) *doubiso*, a double-layered CLB model with isotropically oriented clusters, (2) *simpiso*, a single-layered CLB model with isotropically oriented clusters, (3) *doubori*, a double-layered CLB model with anisotropically oriented clusters, and (4) *simpori*, a single-layered CLB model with anisotropically oriented clusters. These configurations were used because they were shown to produce realistic simulated mammograms under radiologists’ assessment [30]. Additionally, images from the original CLB model *opex99*, proposed by Bochud et al. [42] were employed. The gray levels and pixel value range were set in accordance with Castella et al. [30]. For each of the five canonical SIMs, a GAN was trained on a dataset of 100,000 256×256 images from the SIM.

As discussed in the Introduction, medical image distributions are typically mixed distributions consisting of multiple classes or modes. In order to illustrate the effect of mixing distributions on the identified statistics, a stylized emulation of data coming from two different classes of images was considered. One of the classes consisted of *doubiso* images, which are described above. The other class consisted of *doubiso* images that were first degraded by use of a Gaussian blur followed by low-pass filter ${ℋ}_{LPF}(\cdot )$ with cutoff at half the image bandwidth. Two such multi-class datasets were constructed, one having a 50\50% split and the other having a 95\5% split between the regular and degraded image classes. These two datasets will henceforth be referred to as the *doubiso* 50–50 and *doubiso* 95–5 datasets respectively.

#### 2) B-Mode Ultrasound Speckle Model:

The parameter configurations chosen for the USS SIMs are follows. All images were 256×256 pixels in size with each pixel corresponding to a 100μm×100μm square. The wave speed was set to =1556m/s, the frequency $f_{c}$ was set to 3.5 MHz, the number of cycles within the FWHM was set to $N_{c}=2$, the f-number for the y direction was set to 2 and the f-number in the z direction was set to 3. The frequency and wave speed parameters were set in accordance with [31] while the $N_{c}$ and f-numbers were chosen such that the USS SIM yielded appropriate N values for the given parameters [31]. The ultrasound wave was assumed to be propagating in the x direction. The SND parameter was varied to create four canonical USS SIM datasets. These four datasets corresponded to SND values of 1, 2, 3 and 30 mm^−3^ respectively. The first three values were chosen because they fall in the range of SND values that can be accurately estimated from the image [31]. This is not the case for the *SND-30* SIM that represents fully developed speckle [46]. These four SIMs will henceforth be called (1) *SND-1*, (2) *SND-2*, (3) *SND-3* and (4) *SND-30* respectively.

Additionally, similar to the CLB case, two multi-class datasets were considered. These included (1) a dataset where *SND-2* and *SND-3* were distributed with a 50% – 50% split and (2) a dataset where *SND-2* and *SND-3* were distributed with a 95% – 5% split. Henceforth these datasets will be referred to as the *USS Mixed 50–50* and *USS Mixed 95–5* datasets.

For each of the considered USS SIMs, a GAN was trained using 100,000 images from the SIM. Before training, each ensemble of training images was converted to an unsigned, 8-bit grayscale where 255 corresponds to the top 1% pixel value in the ensemble.

#### 3) The S2V Model:

The S2V was obtained from the 3D VICTRE NBP SOM described in Section II as follows. First, a collection of 1000 3D NBPs was generated using the VICTRE tool [7]. Next, linear attenuation coefficients in cm^−1^ for X-rays of energy 30 keV were assigned to the pixels corresponding to each of the tissue types. This value of the X-ray energy was chosen because of its relevance to breast CT systems [48]. The attenuation values were either directly obtained from literature, or calculated using the mass attenuation coefficient and material density values obtained from literature [49], [50], [51]. Coronal slices were extracted from a central region of an NBP that ranges from 40% through 70% of the distance from the outermost coronal plane to the innermost coronal plane. This was done to avoid extracting slices too close to the chest wall or the nipple. A spacing of 50 pixels was maintained between two slices consecutively extracted from the same NBP. The extracted slices were then downsampled to an image dimension of 512×512, which corresponds to the length scale of 0.4μm per pixel. The described procedure generated a 2D dataset of 130,000 slices, which was used for training a GAN.

StyleGAN2, proposed by Karras et al. [21] was employed as the GAN in all the studies described in this work. All the default parameters and configurations of the StyleGAN2 architecture were kept the same as the the original code base, except for the number of channels in the output image, which was set to 1. The networks were trained using Tensorflow 1.14/Python [52] on an Intel Xeon Gold 5218 CPU and two Nvidia Quadro RTX 8000 GPUs.

### B. Identification and Computation of Evaluation Measures Pertinent to the SIMs

A GAN may learn different types of image statistics to different levels of correctness. Hence, it is important to evaluate GANs using measures based on those statistics that are meaningful and pertinent to the SIM considered. In this study, statistics of image features that are relevant to a specified diagnostic task were chosen as the meaningful evaluation measures. Additionally, statistics deemed useful for assessing the realism of images by radiologists were also used as the meaningful evaluation measures. These statistics were computed from both the “directly simulated” images, i.e. images directly simulated from the canonical SIM, as well as the GAN-generated images.

#### 1) The CLB Model:

The 17 texture features identified by Castella et al. mentioned in Section II have been utilized to improve the clinical realism of CLB images as judged by radiologists [30]. Therefore, these statistics were chosen as the statistics for assessing a GAN trained by use of the CLB SIMs described in Section III-A,. These texture features include those derived from the per-image, gray-level intensity distribution, gray-level co-occurrence matrices (GLCMs) [53], primitives matrices (GLRM), and the neighborhood gray tone difference matrix (NGTDM) [54].

Specifically, the following 17 texture features described by Castella, et al. [30] were computed from each image of the evaluation datasets. Mean, standard deviation, skewness and kurtosis were derived from the per-image gray-level intensity distribution. The texture features energy, entropy, maximum, contrast and homogeneity were computed from the GLCMs. Four features were derived from the primitives matrices (GLRMs), namely, the short primitive emphasis (SPE), long primitive emphasis (LPE), gray level uniformity (GLU), and primitive length uniformity (PLU). The four features derived from the NGTDM [54] were coarseness, contrast, complexity and strength. Various parameter values required for the computation of the texture features, such as the number of gray levels, two-point distances and angles were fixed to the values used in Castella, et al. [30]. The resulting feature data were then used for further analysis in order to summarize trends. Two types of analyses were conducted on the feature data. For the first analysis, the empirical JS divergence between the directly simulated and GAN-generated texture-feature distributions was computed [55]. For the second analysis, principal component analysis (PCA) of the texture-feature data was conducted. The first two principal components of the texture-feature data were selected. An empirical PDF over these two components was computed and plotted for both the directly simulated and GAN-generated texture feature data.

#### 2) B-Mode Ultrasound Speckle Model:

Previous studies have shown that the intensity signal-to-noise ratio (SNR) of USS images is associated with the envelope statistics [56]. In regions of the body such as the liver and the breast, the envelope statistics have been successfully exploited for tissue characterization [56]. Therefore, the SNR was considered to be a statistic pertinent to the USS SIM. Note, however, that this preliminary study does not associate a given speckle model with a tissue type.

The PDF of the ${SNR}^{2}$ estimate of USS speckle can be modeled as a Gaussian distribution centered around the true ${SNR}^{2}$. If the scatterers per resolution cell N follows a Poisson distribution, then one can estimate N using ${SNR}^{2}$. The SNR and N estimate called $\overset{ˆ}{N}$ are defined as [31]:

(2) $$SNR=\frac{\mu_{I}}{\sigma_{I}},\overset{ˆ}{N}=\frac{{SNR}^{2}}{1\;-\;{SNR}^{2}},$$

where $\mu_{I}$ and $\sigma_{I}$ are the mean and standard deviation of the intensity. The SNR and $\overset{ˆ}{N}$ were computed on a per-image basis for both the directly simulated and GAN-generated images by use of the empirically estimated values of $\mu_{I}$ and $\sigma_{I}$ from each image in the test dataset. The JS divergence was used as a measure to summarize the discrepancy between the ${SNR}^{2}$ PDFs of the directly simulated and GAN-generated images.

#### 3) The S2V Model:

Human female breasts can be categorized into four different types based on the relative amount of fat and glandular tissue [47]. It is known that the ratio of the amount of fat compared to the glandular tissue is an important factor impacting the risk of developing breast cancer [57], [58]. This ratio also impacts the effectiveness of screening tests such as mammography in detecting breast masses [47], [57]. Fat and glandular tissue have different linear attenuation coefficients [49], [50], [51]. Therefore, the ratio of fat-to-glandular tissue, denoted as $\rho_{F:G}$, was chosen as a statistic pertinent to the S2V SIM. For the idealized S2V SIM described in Section III-A, the value of $\rho_{F:G}$ corresponding to a thin coronal slice of an NBP was computed as follows. First, the number of pixels F having linear attenuation coefficient values within 1.5% of the linear attenuation coefficient of fat was computed. This thresholding criterion was decided based on the extent of the peak corresponding to fat in the gray-level histogram shown in Fig. 3d. A similar computation was done to obtain the number of pixels G corresponding to the glandular tissue. The fat-to-glandular ratio was then computed as $\rho_{F:G}=F/G$. The linear attenuation coefficient value of fat and glandular tissue are far enough to not confound a simple thresholding-based segmentation scheme required to do the above computation. Hence, the values of F, G and $\rho_{F:G}$ could be estimated accurately both for the directly simulated and GAN-generated images. Using this procedure, $\rho_{F:G}$ was estimated on a per-image basis for both the directly simulated and GAN-generated images. The empirical PDFs of $log{\;\rho }_{F:G}$ computed from both the directly simulated and GAN-generated images were plotted, and the JS divergence between the two PDFs was computed.

Apart from the above-described measures, basic ensemble statistics, such as the histogram of gray level values and the empirical image autocorrelation were computed from directly simulated and GAN-generated images for all the SIMs. As described in Bochud et al. [42], a Papoulis window was used in order to overcome boundary artifacts in the computation of the autocorrelation. The FID score between a directly simulated and a GAN-generated test dataset, as well as two i.i.d. directly simulated datasets was computed. The latter serves as a heuristic noise floor for the FID score for the particular SIM. A pre-trained InceptionV3 network [40] was employed for this purpose. All the evaluation measures were computed using 10,000 directly simulated and GAN-generated images. Other test dataset sizes were examined, and the computed distributions of statistics were found to be qualitatively no different.

## IV. RESULTS

### A. Qualitative Assessment of Images Generated by the GAN

**Fig. 1.**
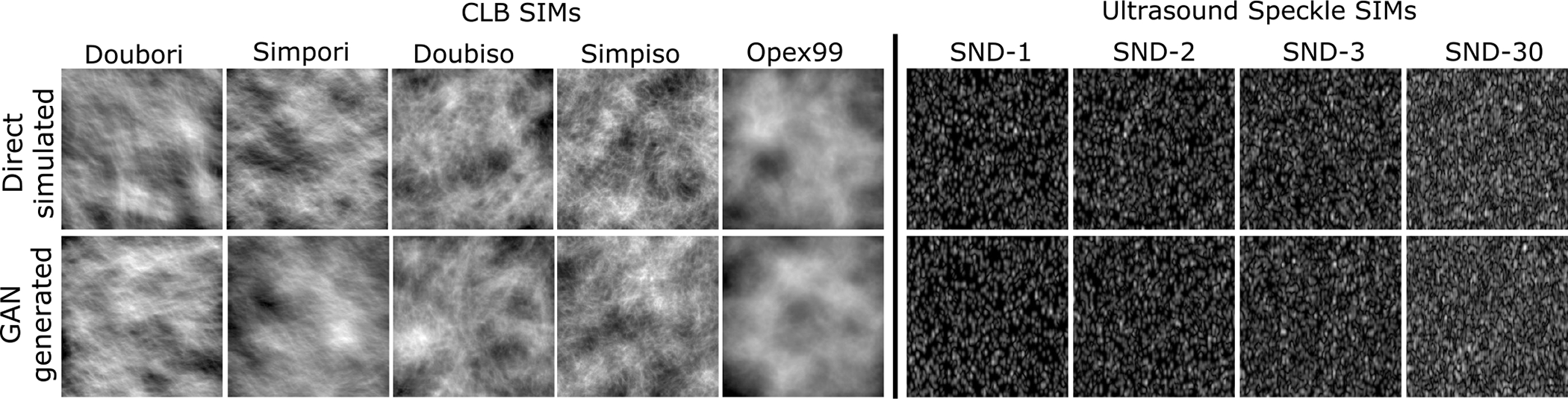
Images simulated from the canonical CLB and USS SIMs and images generated by the GANs trained on images from the SIMs.

**Fig. 2.**
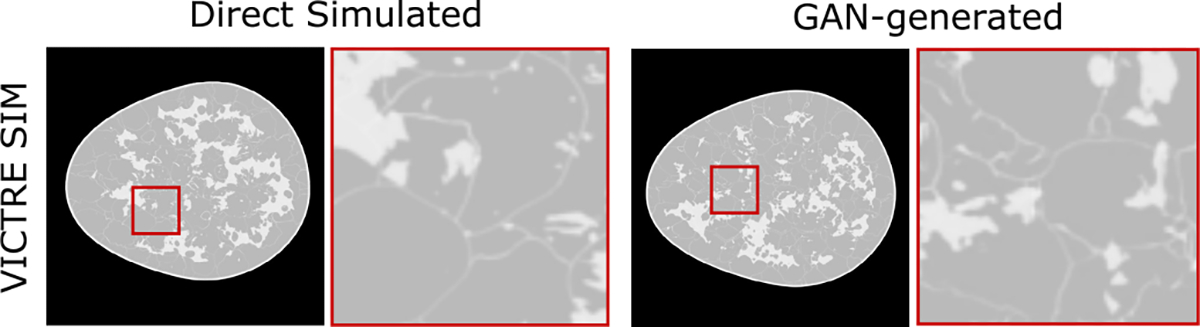
An image simulated from the canonical VICTRE SIM and an image generated by the GAN trained on images from the SIM. In both images, the box outlined in red specifies the location of the zoomed-in region that reveals the fine-scale features such as the ligaments.

Figures 1 and 2 show the images generated by the trained GANs alongside directly simulated images from the training dataset for the single-class CLB, USS and S2V models. It was observed that the directly simulated and the GAN-generated images were visually similar. Note that this is even true for the zoomed-in images of the S2V model shown in Fig. 2. One important thing to note, however, is that some of the ligaments in the GAN-generated images appear broken at certain locations, which is not the case for the directly simulated images. The errors in the images synthesized by GANs were not always easily identified via visual inspection. It should be noted that the images shown in Figures 1 and 2 serve only as examples to demonstrate similarities and differences between the directly simulated and GAN-generated images.

### B. Basic Ensemble Statistics Learned by GANs

**Fig. 3.**
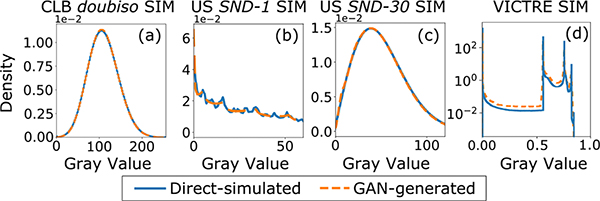
Sample empirical gray level PDFs of direct simulated and GAN-generated images for the three types of SIMs.

Figure 3 shows the ensemble empirical PDF of pixel gray levels for the CLB *doubiso* SIM, the USS *SND-1* and the *SND-30* SIMs, and the S2V SIM, computed from both the directly simulated and GAN-generated images. A close match between these empirical PDFs indicates that the GAN is able to reproduce first-order statistics. The GAN performs similarly for the other CLB SIMs, which have gray-level distributions similar to the ones shown in Fig. 3a. It can be seen that for USS *SND-30* SIM, which represents a fully developed speckle, the GAN reliably reproduces the expected Rayleigh distribution of grayscale values. For the USS *SND-1* SIM, the distribution of grayscale values of the directly simulated images is far from Rayleigh. However, the GAN still recovers this distribution successfully. The pixel-value distributions corresponding to USS *SND-2* and *SND-3* SIMs appear intermediate between the ones shown in Fig. 3b and c.

**Fig. 4.**
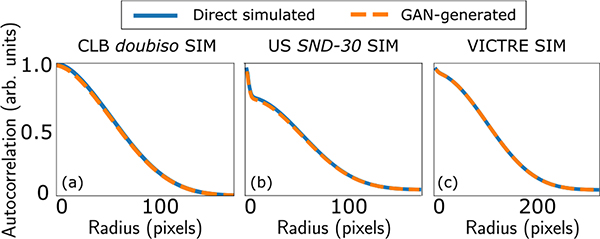
Sample radial profiles of autocorrelation of direct simulated and GAN-generated images for the three types of SIMs.

Fig. 4 shows the radial profile of the image autocorrelation computed using the directly simulated and GAN-generated images for the CLB *doubiso*, USS *SND-1* and S2V SIMs. It can be seen that the GAN was successful in recovering this particular second-order statistic. Similar results were obtained for the other CLB and USS SIMs considered.

### C. Statistics Pertinent to the SIMs Learned by GANs

#### 1) CLB Model:

**Fig. 5.**
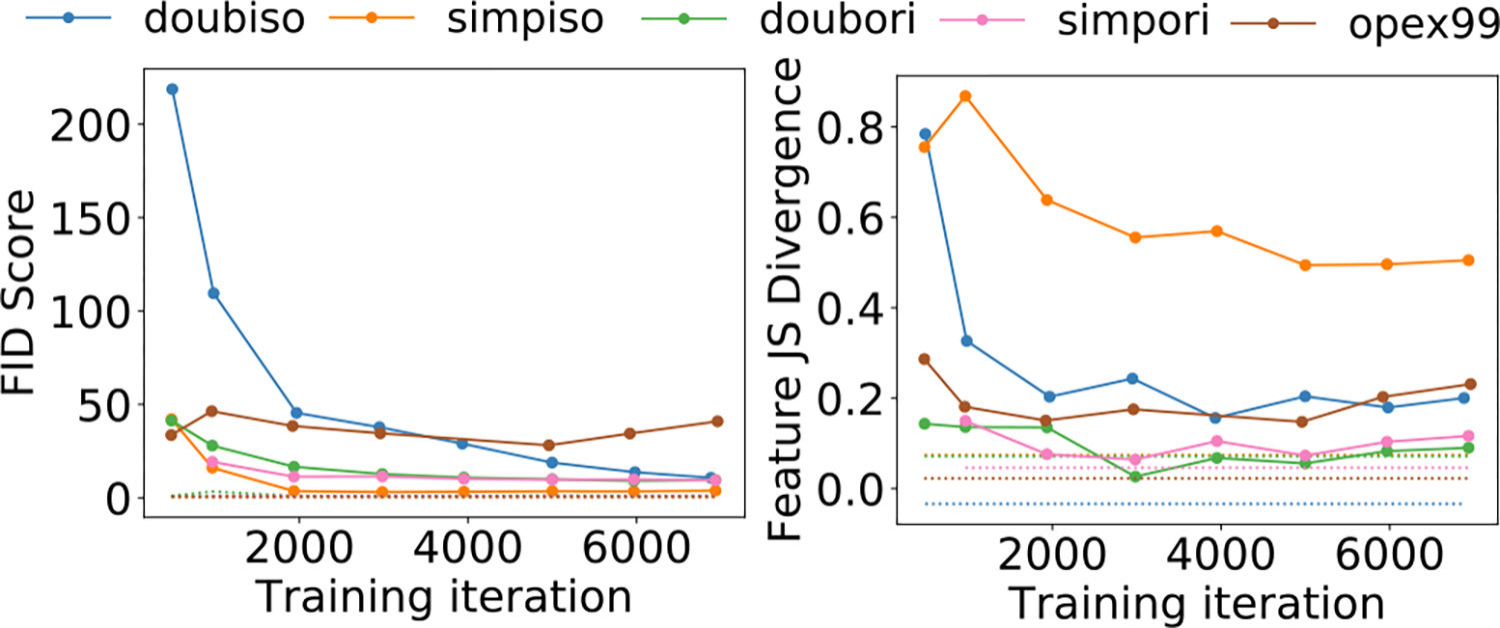
FID and empirical feature JS divergence measures between the real and GAN-generated distrbutions for the five CLB SIMs considered. As a reference measure, the dotted lines shown in the two plots represent the FID and feature-JS divergence computed between two directly simulated datasets instead of a directly simulated and a GAN-generated dataset.

**Fig. 6.**
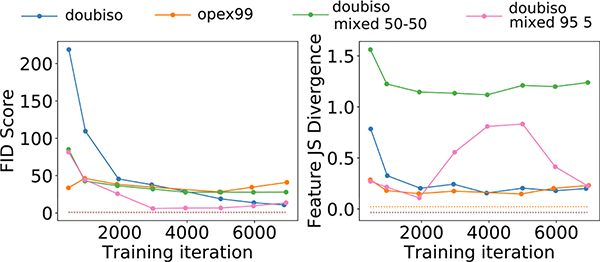
FID and empirical feature JS divergence between the real and GAN-generated distrbutions for *opex99*, *doubiso*, and the two *doubiso-mixed* models. As a reference measure, the dotted lines shown in the two plots represent the FID and feature-JS divergence computed between two directly simulated datasets.

Figure 5 shows the FID as well as the texture feature JS divergence between the directly simulated and GAN-generated distributions as a function of training iteration. In Fig. 6, the FID scores and the feature JS divergences for the *doubiso mixed 50–50* and *doubiso mixed 95–5* datasets are shown along with those for the single class *doubiso* and *opex99* models. As the training progressed, the FID and empirical feature-JS divergence converged for 6 and 7, respectively, out of the 7 SIMs, as revealed in Figures 5 and 6. However, in other cases, these measures either diverged or varied erratically as the training progressed. Furthermore, the high value of the feature JS divergence for the GAN trained on the *doubiso mixed 50–50* model suggests that the GAN was not able to reproduce the meaningful feature statistics as well as the GAN trained on the single class dataset. On the other hand, the FID plot in Fig. 6 shows comparable FID scores for the various SIMs. It does not predict the same trend as the feature JS divergence plots. This suggests that for this specific example, the FID score was ineffective at distinguishing whether multiple modes in the distribution were learned correctly.

**Fig. 7.**
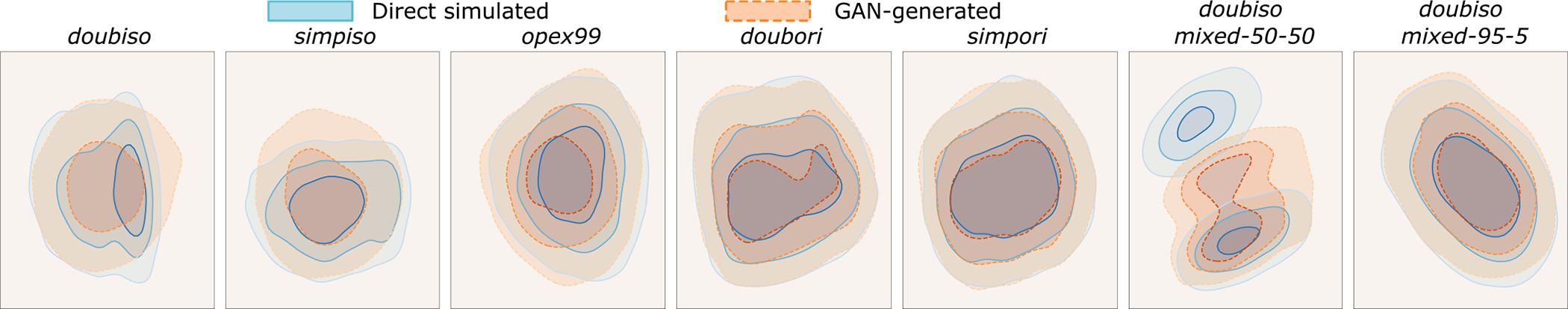
Empirical PDF over the first two principal components of the CLB feature data. The blue and the orange contour plots denote the directly simulated and GAN-generated distributions respectively. For the *opex99*, *simpori* and the *doubiso mixed 95–5* SIMs, the contour lines for the two PDFs overlap, indicating that the GAN learned the PDF over the first two texture feature components well. This was not the case for the other SIMs.

**Fig. 8.**
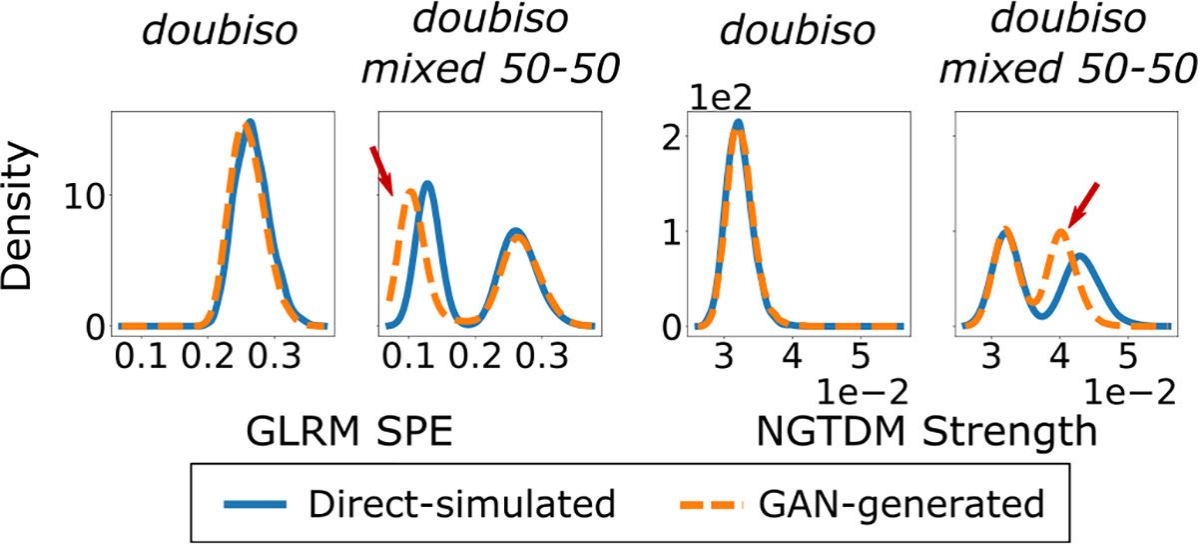
Distributions of per-image GLRM short primitive emphasis (SPE) and NGTDM strength features learned by the GAN for the *doubiso* and *doubiso mixed 50–50* SIMs. The red arrows point to the parts of the distribution corresponding to the degraded class that are learned incorrectly by the GAN.

These findings were further investigated using the principal components of the texture feature data. The procedure for computing these components was described earlier in Section III-B. Figure 7 displays the joint empirical PDF over these components for the directly simulated and GAN-generated images. Note that these texture features were computed on a per-image basis. For most of the CLB SIMs, obvious dissimilarities between the original and learned distributions were observed. This observation is consistent with the trend in the feature JS divergence values shown in Figures 5 and 6, but not with the corresponding FID values. For the *doubiso mixed 50–50* SIM, it can be seen that the GAN failed to correctly learn the distribution of principal NGTDM and GLRM texture components for one of the classes. On further investigation and comparison with the individual texture distributions for the *doubiso* SIM, it was revealed that among others, the GAN failed to learn the per-image GLRM short primitive emphasis (SPE) and the NGTDM strength distributions of the images from the degraded class, as shown in Fig. 8. This was despite the GAN being able to learn ensemble measures such as the FID and basic first- and second-order statistics well.

#### 2) B-Mode Ultrasound Speckle Model:

**Fig. 9.**
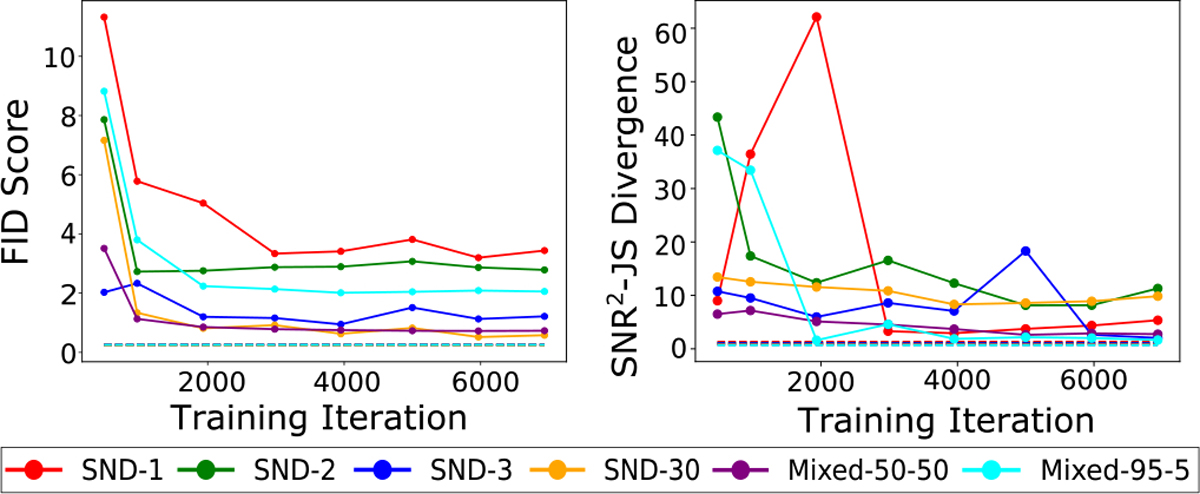
FID and ${SNR}^{2}-JS$ divergence between the real and GAN-generated distributions for *SND-1*, *SND-2*, *SND-3*, *SND-30*, *USS Mixed 50–50* and *USS Mixed 95–5*. The dotted lines shown in the two plots represent the FID and ${SNR}^{2}-JS$ divergence computed between two directly simulated datasets instead of a directly simulated and a GAN-generated dataset.

The empirical JS divergence between the estimated ${SNR}^{2}$ PDFs was computed from the directly simulated and GAN-generated USS images. This quantity will henceforth be referred to as the ${SNR}^{2}-JS$ divergence. The FID score and the ${SNR}^{2}-JS$ divergence as a function of training iteration are shown in Fig. 9. The ${SNR}^{2}-JS$ divergence converges and approaches the noise floor for 5 out of 6 SIMs. However, it behaves erratically at some stage in the training for 2 out of 6 SIMs, even as the FID score for the corresponding SIM converges.

**Fig. 10.**
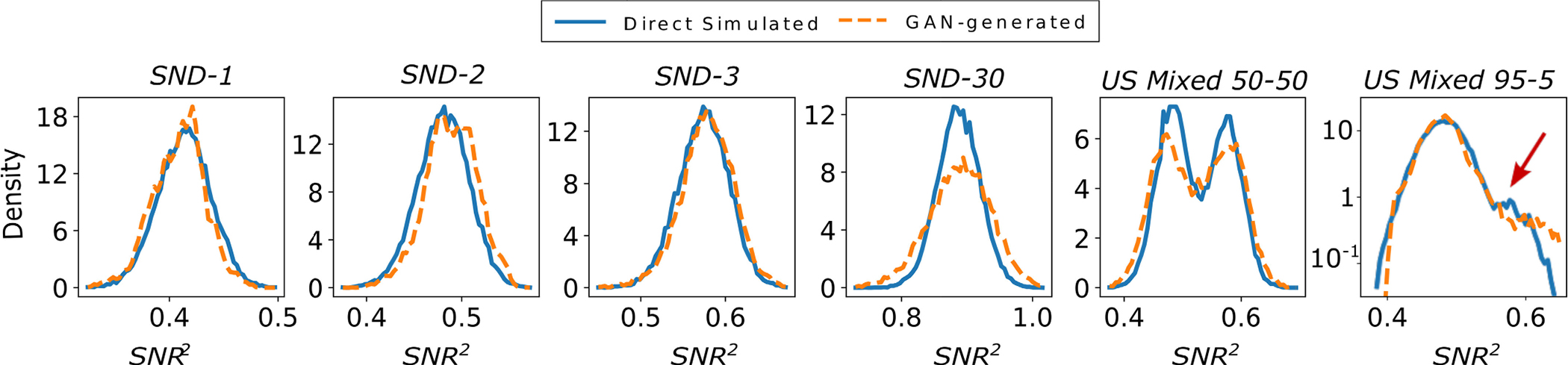
Estimated ${SNR}^{2}$ PDFs of both directly simulated and GAN-generated images for *SND-1*, *SND-2*, *SND-3*, *SND-30*, *USS Mixed 50–50* and *USS Mixed 95–5*. Although the directly simulated and GAN-generated distributions tend to match well, occasionally this is not the case as can be seen in for *SND-30* and *USS Mixed 50–50*. Note that the *USS Mixed 95–5* ${SNR}^{2}$ PDF has the density in log scale with the red arrow pointing to the distribution of the *SND-3* class having 5% prevalence.

In Fig. 10 the estimated ${SNR}^{2}$ PDFs are plotted for both directly simulated and GAN generated USS images. As can be seen the GAN generated images tend to give ${SNR}^{2}$ distributions that somewhat match those of the directly simulated images for the *SND-1*, *SND-2* and *SND-3* SIMs. Since the ${SNR}^{2}$ is theoretically expected to be distributed as a Gaussian for these SIMs [59], each distribution of directly simulated and GAN-generated images was fit to a Gaussian. In Table I the mean and standard deviation of the best fit Gaussian distribution found using least-squares regression are shown in the first two rows. The third row in Table I shows the mean squared error between a given ${SNR}^{2}$ distribution and its Gaussian fit. The results for the mean and standard deviation of the Gaussian fit distributions confirm our visual inspection. The mean values were near perfect matches and so are the standard deviations with the exception of *SND-30*. However, the *MSE* between the GAN-generated empirical ${SNR}^{2}$ PDFs and their Gaussian fits was larger than the *MSE* between the directly simulated empirical ${SNR}^{2}$ PDFs and their Gaussian fits. Finally, the mean estimate of scatterers per resolution cell $\overset{ˆ}{N}$ computed from GAN-generated images was close to that computed from the directly simulated images for all USS SIMs except for *SND-30*. This is expected since the ${SNR}^{2}$ distributions do not match well for the *SND-30* SIM.

In Fig. 9, the FID scores and the ${SNR}^{2}-JS$ divergences can be seen for *USS Mixed 50–50* and *USS Mixed 95–5* SIMs. Interestingly, the *USS Mixed 95–5* SIM has one of the higher FID scores while also having the lowest ${SNR}^{2}-JS$ divergences over training. This could be because even if the ${SNR}^{2}$ distribution over the class having 5% prevalence was not learnt well, it may not significantly impact the JS divergence [60]. Finally, it was observed that the GAN struggled to properly reproduce the directly simulated ${SNR}^{2}$ distributions. In the case of *USS Mixed 50–50*, the ${SNR}^{2}$ distributions of the two classes have greater variance for the GAN-generated images. This results in the GAN producing more images having a value of ${SNR}^{2}$ intermediate between the two classes. For the *USS Mixed 95–5* SIM, the GAN was not able to reproduce the mode corresponding to the class having 5% prevalence in the dataset, as seen in Fig. 10.

#### 3) The S2V SIM:

**Fig. 11.**
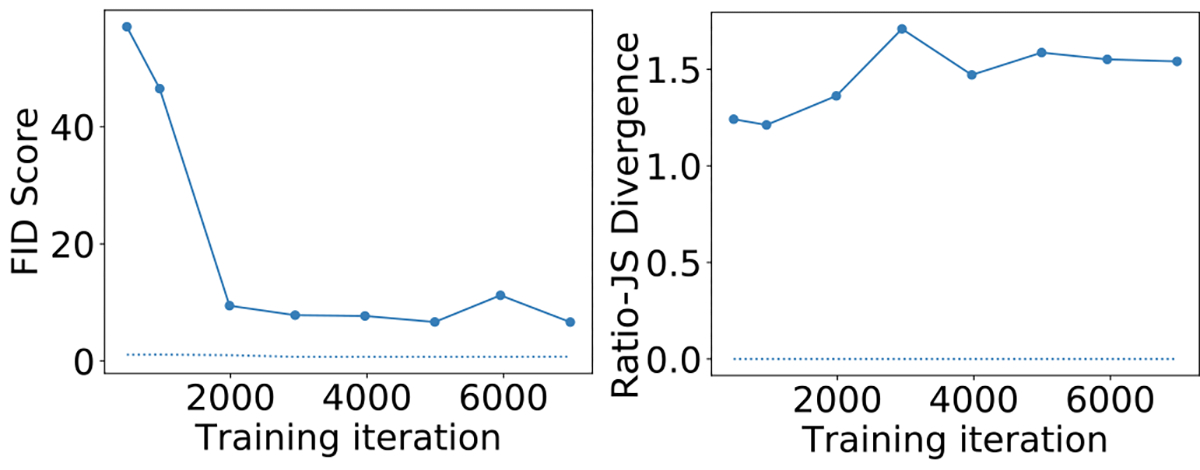
FID and empirical ratio-JS divergence between real and GAN-generated distributions for the S2V dataset. The dotted lines shown in the two plots represent the FID and ratio-JS divergence computed between two directly simulated datasets instead of a directly simulated and a GAN-generated dataset.

**Fig. 12.**
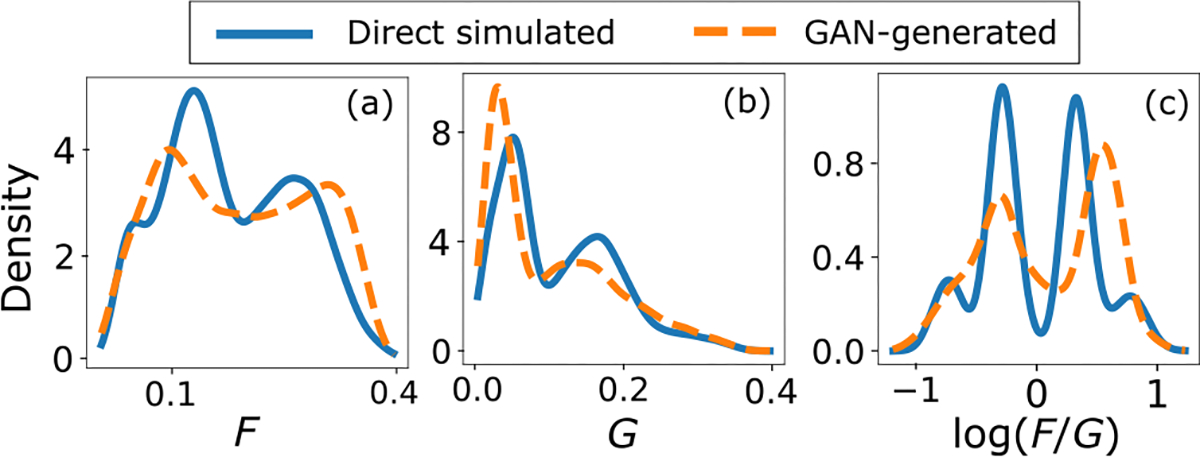
(a-b) The estimated PDF over the per-image number of pixels corresponding to fat and glandular tissue respectively, as a fraction of the total image pixels (denoted by F and G respectively). (c) The estimated PDF over log(F/G).

Figure 11 shows the empirical JS divergence between the empirical PDFs of $log\rho_{F:G}$ computed from the directly simulated and GAN-generated images as a function of the training iteration. This quantity will henceforth be referred to as the ratio-JS divergence. This is displayed alongside the plot of FID score as a function of the training iteration. It was observed that the FID predictably converged as the training progresses. However, the ratio-JS divergence was erratic and did not converge the same way as the FID. Figure 12 shows the empirical PDFs of $log\;\rho_{F:G}$ computed on a per-image basis from the directly simulated and GAN-generated images. The directly simulated distribution clearly shows the four different breast types based on the F:G ratio in their correct clinical prevalence. However, the GAN-generated distribution completely ignored or incorrectly represented many of the breast type modes. This was despite the GAN giving visually appealing images and accurate FID and other basic ensemble metrics.

## V. SUMMARY

The primary objective of this work was to demonstrate medical imaging-relevant methodologies for assessing the statistical information learned by GANs. To accomplish this, we employed SIMs as an enabling technology. GANs have traditionally been evaluated using mathematical or perceptual measures that may not correlate with those statistics that are important with respect to a downstream task. For medical imaging applications, however, it is imperative to understand the capability of a GAN to capture relevant image statistics. Such assessments can lead to the identification of GANs that fail to reproduce important spatial statistics and can guide the development of improved models that do. These assessments can also serve as a precursor to subsequent objective assessments based on signal detection or estimation theory.

The GANs employed consistently produced images that visually appeared realistic, and were able to accurately and consistently reproduce basic statistics such as the intensity histograms and image autocorrelation. It was also observed that although most of the evaluation measures used in this paper converged, they did not necessarily converge at the same rate, and some of them diverged as the training progressed. This demonstrated that despite being commonly used to tune medical image GANs [61], [62], convergence of FID to a low value does not guarantee the correct convergence of the task-relevant statistics. As such, the FID cannot always be used as a proxy for task-relevant measures when, for example, deciding the optimal stopping point for training or choosing the optimal set of hyperparameters or network architectures. Since the FID score measures the Fréchet distance in the feature space of an Inception network trained on the ImageNet dataset, it is not tailored to the specific medical image distribution considered. Additionally, the GAN may learn the distribution of different features to different degrees of fidelity, resulting in different performance rankings when examined by different measures.

From Figures 5, 6 and 9, we note that the FID score and the pertinent metrics *may* give rise to different rank-orderings of the various models. However, the statistical significance of these results remain unproven. This would require computation of confidence intervals for every model, for the all the measures, and the various training iterations considered. This was computationally prohibitive in the current study.

We note that for all the SIMs considered, the GAN-generated images retained potentially impactful errors in individual image realizations in some of the image features identified. These errors impacted the empirical PDFs of these features computed from GAN-generated images. Critical problems such as mode-dropping and merging of multiple classes or modes were observed due to these errors. This was despite the GAN producing excellent agreement with the directly simulated distribution in terms of ensemble measures, such as the FID and basic first- and second-order statistics. This demonstrated that a GAN trained on medical images may synthesize images with errors while still yielding accurate ensemble statistics.

These observations point to the need for choosing evaluation measures that are (1) meaningful and pertinent to the SIM considered, (2) are motivated by a downstream task, and (3) are sensitive to the important aspects of a medical image distribution, such as multiple modes. Formulating such evaluation measures requires significant effort. However, it opens up the possibility of evaluating GANs in terms of those statistics that influence task-performance.

A full-fledged task-based assessment of GANs is important but remains a topic for future research. While task-based measures are of ultimate interest, such measures do not directly provide insights into failure modes or image characteristics that are not reliably learned by a GAN. When evaluating emerging technologies such as GANs, understanding such issues is critical. Also, when a GAN is employed for medical image synthesis, the resulting images may be employed for different tasks, and good performance on one task does not guarantee good performance on another.

This work presents a framework for comparing distributions of relevant image statistics that have long been known to be clinically relevant for a wide range of tasks [43], [47]. Such studies can enable the triage of GAN models that do not faithfully capture important statistics of medical images and will accelerate their refinements before significant efforts are spent on task-based assessments.

0 On the other hand, image statistics, such as the ones described in this paper have long been known to be clinically relevant for a wide range of tasks [43], [47]. Therefore, the presented evaluation framework could be used to triage GAN models before rigorous task-based assessments can be performed.

The presented study possesses certain limitations. For example, as mentioned before, uncertainty quantification of certain measures considered could be prohibitively expensive computationally, and hence has been excluded from the study. Therefore, we do not make any claims about the difference in the rank-ordering of the FID and JS-divergence curves, though such a difference seems plausible from our results. Additionally, the proposed methodology uses domain-specific metrics to assess GANs. For other SIMs not considered, these metrics would need to be identified.

This study employed the StyleGAN2 architecture, since it has been shown to consistently produce realistic images when trained on a wide variety of datasets. However, the proposed analysis could readily be performed on other types of generative models. It was not possible to comprehensively tune the large number of hyperparameters associated with the Style-GAN2 architecture. Hence, it is possible that a StyleGAN2 with an optimal parameter configuration is able to correctly learn the identified statistics. Canonical SIMs that produce simulated medical images enable us to examine the behavior of the GAN under a controlled setting with different parameter configurations. Nevertheless, evaluating GANs trained on real medical images remains a topic for future investigation.

## Figures and Tables

**TABLE I:** A Table Showing the Mean μ and Standard Deviation σ of the Gaussian Fit Curve for both Directly Simulated and GAN-Generated ${\mathrm{SNR}}^{2}$ Distributions, the Mean Squared Error (MSE) Between the Gaussian Fit and Their Respective ${\mathrm{SNR}}^{2}$ Distributions and the Mean Scatterers per Resolution Cell Estimate $\hat{N}$ of Both Directly Simulated (D.S.) and GAN-Generated (G.G.) Images. The 95% Confidence Intervals for the Above Results Are Orders of Magnitude Smaller Than the Significant Digits Shown and Are Thus Omitted.

	*SND-1*		*SND-2*		*SND-3*		*SND-30*	
	D.S.	G.G.	D.S.	G.G.	D.S.	G.G.	D.S.	G.G.
μ	0.415	0.411	0.482	0.493	0.576	0.581	0.888	0.892
σ	0.024	0.024	0.027	0.028	0.030	0.030	0.032	0.047
*MSE* (10^−4^)	2.929	20.923	3.022	24.369	2.554	3.328	1.316	2.602
$\hat{N}$	0.713	0.699	0.936	0.975	1.369	1.394	7.544	9.784
